# Kaurenoic acid is a potent inhibitor of SARS-CoV-2 RNA synthesis, virion assembly, and release *in vitro*

**DOI:** 10.3389/fmicb.2025.1540934

**Published:** 2025-05-09

**Authors:** Igor Andrade Santos, Victoria Riquena Grosche, Natasha Marques Cassani, Rodrigo Cássio Sola Veneziani, Gustavo Lima Ribeiro, Jairo Kenupp Bastos, Nilson Nicolau-Junior, Andres Merits, Carlos Henrique Gomes Martins, Mark Harris, Ana Carolina Gomes Jardim

**Affiliations:** ^1^Institute of Biomedical Sciences, Federal University of Uberlândia, Uberlândia, Brazil; ^2^Faculty of Biological Sciences, School of Molecular and Cellular Biology, University of Leeds, Leeds, United Kingdom; ^3^Institute of Biosciences, Humanities and Exact Sciences, São Paulo State University, São Jose do Rio Preto, Brazil; ^4^Nucleus of Research in Sciences and Technology, University of Franca, Franca, Brazil; ^5^Faculty of Pharmaceutical Sciences of Ribeirão Preto, University of São Paulo, Ribeirão Preto, Brazil; ^6^Institute of Biotechnology, Federal University of Uberlândia (UFU), Uberlândia, Brazil; ^7^Institute of Bioengineering, University of Tartu, Tartu, Estonia

**Keywords:** antiviral, COVID-19, natural compounds, SARS-CoV-2, phytotherapeutics

## Abstract

**Introduction:**

Severe acute respiratory syndrome coronavirus 2 (SARS-CoV-2), which is responsible for the coronavirus disease 2019 (COVID-19) pandemic, continues to pose global health challenges despite the availability of approved vaccines and antiviral drugs. The emergence of new variants of SARS-CoV-2 and ongoing post-COVID complications necessitate continuous exploration of effective treatments. Kaurenoic acid (KA) is a tetracyclic diterpenoid isolated from plants of the *Copaifera* genus and has been previously recognized for its anti-inflammatory, antibacterial, antifungal, and antitumor properties. However, there is a lack of knowledge about the *in vitro* effects of KA on viruses. Here, we evaluated its effect on SARS-CoV-2 replication for the first time.

**Methods and Results:**

KA demonstrated a high selective index of 16.1 against SARS-CoV-2 and robust effectiveness against the B.1.617.2 (Delta) and BA.2 (Omicron) variants. Mechanistically, KA was shown to impair the post-entry steps of viral replication. In a subgenomic replicon system, we observed a decrease in viral RNA synthesis in different cell lines. Using an infectious virus, a larger reduction in the release of SARS-CoV-2 virions was observed. We suggest that KA interacts with SARS-CoV-2 proteases through molecular docking.

**Conclusion:**

In conclusion, KA emerges as an inhibitor of SARS-CoV-2 proteases and, consequently, its replication cycle. It could be a good candidate for further investigation in clinical assays against SARS-CoV-2 infection.

## Graphical abstract

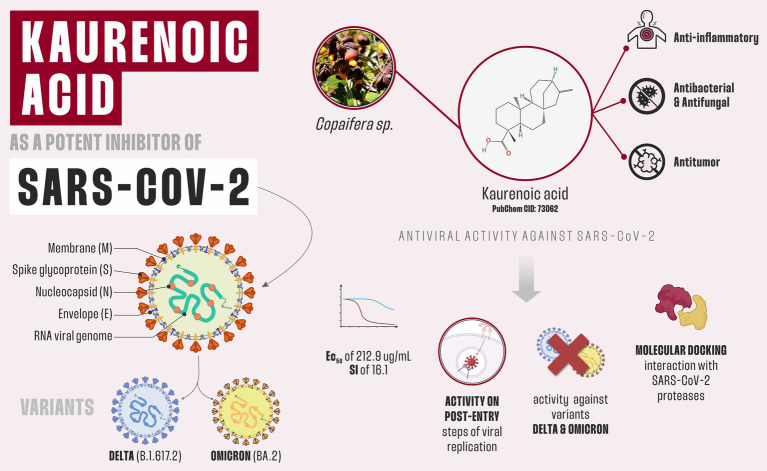

## 1 Introduction

*Severe acute respiratory syndrome coronavirus 2* (SARS-CoV-2) is the etiological agent of Coronavirus disease 2019 (COVID-19) and belongs to the genus *Betacoronavirus* in the *Coronaviridae* family (Hillary and Ceasar, 2023). SARS-CoV-2 is transmitted mainly by respiratory droplets, leading to asymptomatic cases or causing symptoms such as cough, headache, and fever. However, numerous COVID-19 patients develop a severe chronic disease with dyspnea, hypoxemia, and acute respiratory distress syndrome (ARDS), which eventually can lead to death. Furthermore, COVID-19 is also characterized as an extrapulmonary disease with kidney and liver injury, gastrointestinal symptoms, arrhythmias, and coagulopathy (Trougakos et al., 2021; Hillary and Ceasar, 2023).

As of mid-2024, 10 vaccines used for prophylaxis against COVID-19 have received approval from the U.S. Food and Drug Administration (FDA), including vaccines manufactured by Sinovac-Coronavac, AstraZeneca/Oxford, Janssen, Pfizer/BioNTech, and Moderna (COVID-19 Vaccines for 2024-2025 | FDA). In addition, three directly acting antiviral drugs have been approved for COVID-19 treatment: polymerase inhibitors Remdesivir and Molnupiravir as well as the protease inhibitor Paxlovid® (Beigel et al., 2020; Fischer et al., 2022; Jayk Bernal et al., 2022). Even though these drugs have played an important role in COVID-19 management, they were not designed to address the SARS-CoV-2 replication directly. In addition, they can induce viral resistance and adverse effects and are prescribed only for severe cases of COVID-19, which do not always result in a reduction in the duration of infection, hospitalization time, and/or mortality (Amstutz et al., 2023). Additionally, COVID-19 still burdens health systems worldwide due to the emergence of variants of SARS-CoV-2 with mutations that result in increased transmissibility, immune evasion, and waning of immunity obtained through vaccination and/or previous SARS-CoV-2 infection (Lippi et al., 2022). Altogether, these might interfere with the efficacy in controlling the current pandemic. In the worst scenario, patients who have recovered from COVID-19 frequently develop post-COVID syndrome, in which symptoms can last for 9 or more months. The symptoms/complications described so far encompass neurological effects (brain fog, loss and/or impairment in vision), thromboembolism, cardiac arrhythmia, infertility, deregulation of the menstrual cycle, and the thyroid gland (Lee et al., 2024; Maruki et al., 2024). In this context, identifying novel molecules, especially those that are compatible with the already-approved ones, is an attractive approach for discovering new anti-SARS-CoV-2 drugs.

Natural products (NPs) are important leads in developing drugs against a diversity of chronic and infectious diseases. The diversity of scaffolds and the structural complexity found in NPs are unparalleled (Butler, 2005; Newman and Cragg, 2020). The importance and impact of NPs in the treatment of COVID-19 are evidenced by the fact that, to date, they continue to generate anti-SARS-CoV-2 candidates. Among them, the *Marantodes pumilum* extract, SKF7, was described as a SARS-CoV-2 inhibitor by impacting 3CLpro activity. Additionally, the arylnaphthalene lignan 6′-hydroxy justicidin B, isolated from *Justicia procumbens*, reduced viral replication both *in vivo* and *in vitro* (Yoo et al., 2024), while xanthohumol and its derivatives from *Humulus lupulus* were characterized as SARS-CoV-2 protease inhibitors (Herzog et al., 2024). Thus, investigating molecules isolated from natural sources reveals promising therapeutic potential.

Trees belonging to the genus *Copaifera* are an attractive source of bioactive molecules. The genus comprises 72 recognized species of which over 20 are found in Brazilian flora (Veiga et al., 2007; Leandro et al., 2016; Damasceno et al., 2019). NPs isolated from this genus showed good activity against ulcers, inflammation, leishmaniasis, and gonorrhea (Furtado et al., 2018), and therefore these plants are frequently used in traditional medicine. Kaurenoic acid (KA) is a tetracyclic diterpenoid commonly found in *Copaifera* spp., and it is known for its anti-inflammatory, antibacterial, antifungal, and antitumor properties (Leandro et al., 2016; Matos et al., 2018). Our group isolated and characterized KA from *Copaifera oleoresin*, and its derivatives against human breast carcinoma cell line (Simão et al., 2016; Carneiro et al., 2020); however, to the best of our knowledge, little is known about the antiviral properties of KA. Therefore, here we evaluate and characterize the potential of KA to inhibit SARS-CoV-2 replication.

## 2 Methods

### 2.1 Cell culture and the compound

Human adenocarcinoma alveolar basal epithelial cells expressing ACE2 and TMPRSS2 receptors (A549-AT, National Institute for Biological Standards and Control [NIBSC], United Kingdom, #101004), African green monkey kidney cells expressing ACE2 (Vero-E6-ACE2, NIBSC, United Kingdom, #101001), and baby hamster kidney cells (BHK-21, ATCC #CCL-10) were cultivated in Dulbecco’s modified Eagle’s medium (DMEM; Sigma–Aldrich, Gillingham, United Kingdom) supplemented with 100 U/mL penicillin (Gibco Life Technologies, Thermo-Fisher Scientific, Paisley, United Kingdom), 100 mg/mL streptomycin (Gibco Life Technologies, Thermo-Fisher Scientific, Paisley, United Kingdom), 1% (v/v) non-essential amino acids (Gibco Life Technologies, Thermo-Fisher Scientific, Paisley, United Kingdom) and 10% (v/v) fetal bovine serum (FBS; Hyclone, Logan, UT, United States) at 37°C in a humidified 5% CO_2_ incubator. A549-AT cells were cultivated in the presence of Geneticin (G418, Invitrogen, Paisley, United Kingdom) and Hygromycin B (Sigma–Aldrich, Gillingham, United Kingdom) at 2 mg/mL and 200 μg/mL, respectively (Oliveira et al., 2024).

KA was first isolated by our group in 2016 from *C. oleoresin* (Simão et al., 2016), and to perform the assays described herein, a re-isolation was carried out following the previously described methods. Briefly, *C. oleoresin* (150 g) was chromatographed over silica gel 60H (Merck, art. 7736) using vacuum liquid chromatography (VLC) (Pelletier et al., 1986) with increasing amounts of 20% ethyl acetate in *n*-hexane as eluent, thus yielding six fractions (F1–F6). Thin-layer chromatography (TLC) analysis and comparison with an analytical standard revealed that KA was present as the major compound in fraction 2 (23.3%). Then, F2 was submitted to another VLC using silica gel 60H (Merck, art. 7736) with increasing amounts of 3% ethyl acetate in *n*-hexane as the eluent, resulting in 10 fractions (F2.1–F2.10). After TLC analysis, it was noticed that KA was present in the seventh fraction (F2.7; 11.4 g), thus yielding 5.7 g of this compound after washing with cold methanol. The compound identification was confirmed through spectroscopic analysis, with a purity between 95% and 98% confirmed by integration of peak areas in the ^1^H nuclear magnetic resonance (^1^H NMR) spectrum. This method was based on a previous method published by our group (Nascimento and Oliveira, 2001) and supported by the ^1^H NMR studies (Cushman et al., 2014). The isolated compound was dissolved in dimethyl sulfoxide (DMSO, 99.9% v/v). KA NMR data: ^1^H-NMR (CDCl_3_, 400 MHz): δ = 4.79 (1H,s, H-17b) and 4.73 (1H, s, H-17a); 2.63 (1H, s, H-13); 1.24 (3H, s, H-18); 0.94 (3H,s H-20). ^13^C-NMR (CDCl_3_,100 MHz): δ = 40.7 (C-1), 19.1 (C-2), 37.8 (C-3), 43.8 (C-4), 57.1 (C-5), 21.9 (C-6), 41.3 (C-7), 44.3 (C-8), 55.1 (C-9), 39.7 (C-10 and C-14), 18.5 (C-11), 33.2 (C-12), 43.9 (C-13), 49.0 (C-15), 155.9 (C-16), 103.0 (C-17), 29.0 (C-18), 184.7 (C-19), and 15.6 (C-20) as shown in Supplementary Figure 1.

The purity of KA was determined through high-performance liquid chromatography with diode-array detection (HPLC-DAD) analysis using the following conditions: Shim-pack series CLC-ODS column (25 cm × 4.6 mm i.d., 5 μm; Shimadzu, Milton Keynes, United Kingdom) using 90% of acetonitrile in water and 0.1% acetic acid (v/v) at 1.0 mL·min^−1^ as the mobile phase. The column temperature was kept at 40°C, and the injection volume was 20 μL. The acquisition wavelength was 201 nm, and the analysis time was 20 min. KA concentration was 100 μg/mL, and the concentration of usnic acid, internal standard (IS), was 20 μg/mL. Retention times of KA were 8,060 min and of the IS was 12,651 min, and the relative retention time of KA was determined as t_KA_/t_I.S_ = 0,637 (Supplementary Figure 2). The analysis was repeated 3 times; the isolated compound was then dissolved in dimethyl sulfoxide (DMSO, 99.9% v/v), and used at a final concentration of DMSO 0.1% in all assays.

Molnupiravir (*β*-D-*N*4-hydroxycytidine) was purchased from Sigma–Aldrich (EIDD-1931; SML2872-5MG) and used at 10 μM as the positive control since it is widely described as a coronavirus inhibitor (Teli et al., 2023).

### 2.2 Wild-type SARS-CoV-2 variants

The viruses were isolated and characterized at The Francis Crick Institute (United Kingdom) and kindly provided by Prof. Stephen Griffin at the University of Leeds. Three variants were used: the parental Wuhan-like (hCov-119/England/02/2020, GISAID access number: EPI_ISL_407073); B.1.617.2 (Delta) (MS066352H, GISAID access number: EPI_ISL_1731019), and BA.2 (Omicron) (hCoV/England/FCI-179/2022) (Shawe-Taylor et al., 2024). The viruses were amplified in Vero E6-ACE2 cells, their titers were determined using the 50% tissue culture infectious dose (TCID_50_) method, and calculated by the Spearman–Kärber algorithm as described (Hierholzer and Killington, 1996).

### 2.3 Rescue of recombinant SARS-CoV-2 harboring mCherry marker

A total of 1 μg of the pCCI-4K-SARS-CoV-2-mCherry plasmid, harboring infectious complementary DNA (cDNA) of the virus under the control of the CMV promoter (Rihn et al., 2021) was used to transfect BHK-21 cells (3 × 10^5^ cells/well in a 6-well plate) using the Lipofectamine 2000 reagent according to the manufacturer’s (Thermo-Fisher Scientific, United Kingdom) protocol. After 3 days, the supernatant was collected and transferred to A549-AT cells grown in a T75 cm^2^ flask, and cells were incubated until complete cell lysis was observed; then the supernatant was harvested as P0 stock. The infectious titer of the recombinant virus was determined as described above.

### 2.4 Dose–response assay

A549-AT cells were seeded into 96-well plates at a density of 1 × 10^4^ cells *per* well. Then, 24 h later, cells were treated with 2-fold serial dilutions of KA (0.18–200 μg/mL) in the presence or absence of SARS-CoV-2-mCherry at a multiplicity of infection (MOI) of 0.1. Cell viability was measured using 3-(4,5-dimethylthiazol-2-yl)-2,5-diphenyl tetrazolium bromide—also known as MTT (Sigma–Aldrich, United Kingdom) method. Briefly, after 24-h treatment, the medium containing the compound was removed, and MTT at a final concentration of 1 mg/mL was added to each well. Cells were incubated for 30 min, after which MTT solution was replaced with 100 μL of DMSO to solubilize the formazan crystals. The absorbance was measured at 570 nm on a FLUOstar OPTIMA microplate reader (FLUOstar OPTIMA, BMG LABTECH, Ortenberg, DE). To analyze viral inhibition, the plates were placed on the IncuCyte® S3 Live-Cell Analysis System IncuCyte® S3 Live-Cell Analysis System (Sartorius AG, Göttingen, DE); incubated for 24 h, and the red fluorescence light was observed at a 10 × objective. The images were recorded and analyzed employing the basic analyzer from the IncuCyte S3 system to obtain the total integrated intensity of the fluorescence (relative cell unit [RCU] × μm^2^/well). Impact on viral replication and cell viability was calculated according to the equation (T/C) × 100%, in which T and C represented the optical density of the compound-treated and control (DMSO-treated) wells, respectively (De Oliveira et al., 2020; Santos I. A. et al., 2021). For the establishment of effective concentration 50% (EC_50_) and cytotoxic concentration 50% (CC_50_) values, the data were transformed into Log(X), where X is the concentration, and submitted to a non-linear regression with four parameters in variable slope. To confirm the best concentration to perform further assays, KA was added to the cells in the presence or absence of SARS-CoV-2-mCherry in nine different concentrations ranging from 3 to 27μg/mL. Cell viability and viral replication were assessed as previously described.

### 2.5 Time of addition assays

The time of the addition assay was conducted as previously reported (Santos I. A. et al., 2021; Santos et al., 2022; Grosche et al., 2023). Briefly, A549-AT cells were seeded in 96-well plates at a density of 1 × 10^4^ cells *per* well 24 h prior to infection and treatment. In all assays, SARS-CoV-2-mCherry replication was assessed by the red fluorescence light in a 10 × objective, and the photos were analyzed employing the basic analyzer from the IncuCyte S3 system by the collection of total integrated intensity of the fluorescence (RCU × μm^2^/well).

For the pretreatment assay, cells were treated with KA for 1 h prior to the viral infection, extensively washed with phosphate-buffered saline (PBS), and infected with SARS-CoV-2-mCherry at an MOI of 0.1 for 1 h. Next, cells were washed with PBS to remove unbound viruses and incubated with fresh growth medium for 24 h. In the entry inhibition assay, cells were infected using a medium containing KA and a virus at an MOI of 1 for 1 h, washed with PBS, and incubated with fresh medium for 24 h. The virucidal activity of the compound was assessed using the same setting, except that the inoculum containing KA and virus at an MOI of 5 was incubated for 1 h before it was added to the cells. The impact of the compound on the attachment step was analyzed using the same setting as in the entry inhibition assay, except that cells were incubated with virus and compound at 4°C. In an internalization assay, the incubation at 4°C was followed by the incubation at 37°C for 30 min. In a post-entry assay, cells were infected with SARS-CoV-2-mCherry at an MOI of 0.1 for 1 h, washed extensively with PBS, and incubated in a compound-containing medium for 24 h.

### 2.6 Virion formation and release assay

Cells were infected with SARS-CoV-2 Wuhan-like (hCov-119/England/02/2020) at an MOI of 0.1 and treated with KA for 24 h, with DMSO 0.1% used as an untreated control. Then, supernatant and cell lysate from both infected-treated and infected-non-treated wells were collected separately, and total RNA was extracted using TRIzol® reagent (Invitrogen, Waltham, MA, United States) as previously described (Grosche et al., 2023). The cDNA was synthesized using LunaScript® RT SuperMix Kit (New England Biolabs, Hitchin, United Kingdom) according to the manufacturer’s guidelines. The cDNA was quantified using a NanoDrop One/OneC (Thermo-Fisher Scientific, Paisley, United Kingdom). Reverse-transcription quantitative polymerase chain reaction (RT-qPCR) was conducted using 1 μL of cDNA/reaction, primers against SARS-CoV-2 ORF1 (Forward: 5′GACCGAAAGGTAAGATGGAG3′, Reverse: 5′AAATCGCCCGTCTGCCATGAAG3′, designed by the authors) and GoTaq® qPCR Master Mix GoTaq® qPCR Master Mix (Promega, Southampton, UK). The thermal cycler parameters were initial denaturation at 95°C for 10 min, followed by 40 cycles of 95°C for 15 s and 60°C for 1 min. The cycle threshold (Ct) was analyzed, and the SARS-CoV-2 RNA copy numbers were determined based on comparison with the standard curve and expressed on a log10 scale. The standard curve was performed by diluting cDNA from a SARS-CoV-2-mCherry supernatant sample at 10^6^ plaque-forming units (pfu)/mL in a 10-fold serial dilution. For the total RNA isolated from lysates of infected cells, the quantification of viral RNA copy numbers was performed as described above, and the obtained values were normalized to the copy numbers of mRNA of the housekeeping gene GAPDH determined using corresponding primers. Every RT-qPCR assay was performed in duplicate and included both negative (nuclease-free water) and positive (SARS-CoV-2-mCherry cDNA) controls.

### 2.7 Analysis of replication of SARS-CoV-2 subgenomic replicon in BHK-21 and A549-AT cells

The SARS-CoV-2 subgenomic replicon plasmid pCCL-4K-SARS-CoV-2-Repl-PL-NLuc employed in this study has a deletion of the region encoding for spike, membrane, and accessory proteins 3a, 3b, E, 6, 7a, and 7b (positions 21,565–28,124 of the SARS-CoV-2 Wuhan strain genome). The expression of replicon-competent RNA, which contains the gene of a nanoluciferase reporter, is driven by cytomegalovirus promoter (CMV). BHK-21 cells were seeded in 48-well plates at 5 × 10^4^ cells/well, while A549-AT cells were seeded at 3 × 10^4^ cells/well. After 24 h, cells were transfected with 600 ng of the plasmid *per* well, and after 4 h, treated with KA at 10 μg/mL; DMSO 0.1% was used as vehicle control, and Molnupiravir (*β*-D-*N*4-hydroxycytidine) at 10 μM as positive control (Teli et al., 2023). After 72 h incubation, cells were lysed with a passive lysis buffer, and luminescence levels were quantified with the Nano-Glo® Luciferase Assay System (Promega, Southampton, UK). Cell viability assays were carried out simultaneously. Viral replication and cell viability were calculated according to the equation (T/C) × 100%, in which T and C represented the optical density of the treated well and control groups, respectively.

### 2.8 Rescue of SARS-CoV-2 infectious cDNA chimeras expressing variants of concern spikes

To assess the effect of KA against the Delta and Omicron variants, the SARS-CoV-2-mCherry infectious clone (based on the Wuhan strain) was modified by the replacement of the spike gene sequence with that from the variants B.1.617.2 (Delta) or BA.2 (Omicron). The rescue of the viruses was carried out as described above. For the antiviral screening, KA was added to the medium containing the SARS-CoV-2-mCherry-S-Delta or SARS-CoV-2-mCherry-S-Omicron viruses in an amount necessary to achieve infection at an MOI of 0.1. After, samples were added to the A549-AT cells and the total integrated intensity of the fluorescence (RCU × μm^2^/well) was analyzed using the IncuCyte S3 microscope 24-h post-infection.

### 2.9 Antiviral screening with SARS-CoV-2 wild-type variants

For the antiviral assays employing the wild-type SARS-CoV-2 Wuhan-like, SARS-CoV-2 Delta (B.1.617.2), and Omicron (BA.2) variants A549-AT cells were seeded in 12-well plates at a density of 8 × 10^5^ cells/well, and infected with each of virus at a MOI of 0.1 in the presence of KA at 50 μg/mL for 24 h. The supernatant was collected, and the infectious titer was determined using the TCID_50_ method. DMSO 0.1% was used as a negative control.

### 2.10 Molecular docking interaction KA with PL^pro^ and M^pro^

The potential modes of interaction between the ligand KA and SARS-CoV-2 proteases, the three-dimensional (3D-)structures of the papain-like protease (PL^pro^) and the main protease (M^pro^) were collected from the RCSB Protein Data Bank (RCSB PDB) using the identities (IDs) 7CMD and 7CB7, respectively, or from their original manuscripts (Gao et al., 2021). Then, both proteins were screened using the Genetic Optimization for Ligand Docking (GOLD) docking program and scored by the CHEM-piecewise linear potential (CHEM-PLP; Protein–ligand Docking with GOLD | CCDC cam.ac.uk). The binding sites were identified and set by 6Å around known inhibitors associated with these complexes, GRL0617 (PDBid:7CMD) and GC376 (PDBid:7CB7), to perform the docking approach. Ten GA runs were conducted for each protein-ligand docking, with GA settings set to 200% for ligand pose search efficiency. The best-scoring complex of each docking was selected for further analysis. KDeep tool, a protein-ligand affinity predictor based on deep convolutional neural networks (DCNNs), was used to find the predicted binding energy (dG) and ligand efficiency of the docked poses (Jiménez et al., 2018).

### 2.11 Statistical analysis

Antiviral assays were performed with a minimum of three biological replicates, each carried out in quadruplicate. All the data were analyzed for the normality and lognormality tests. Differences between control *vs*. treated samples, control vs. Molnupiravir, or treated samples vs. Molnupiravir were compared using Student’s t-tests or one-way analysis of variance (ANOVA) for time-of-addition, release, and antiviral assay with chimeras and SARS-CoV-2 variants. *p*-values below 0.05 (indicated by asterisks) were considered statistically significant. For the establishment of effective concentration 50% (EC_50_) and cytotoxic concentration 50% (CC_50_) values, data were transformed into Log(X), where X is the concentration, and submitted to a non-linear regression with four parameters in variable slope. All analyses were performed using GraphPad Prism 9 software.

## 3 Results

### 3.1 KA impairs SARS-CoV-2 replication

**Figure 1:**
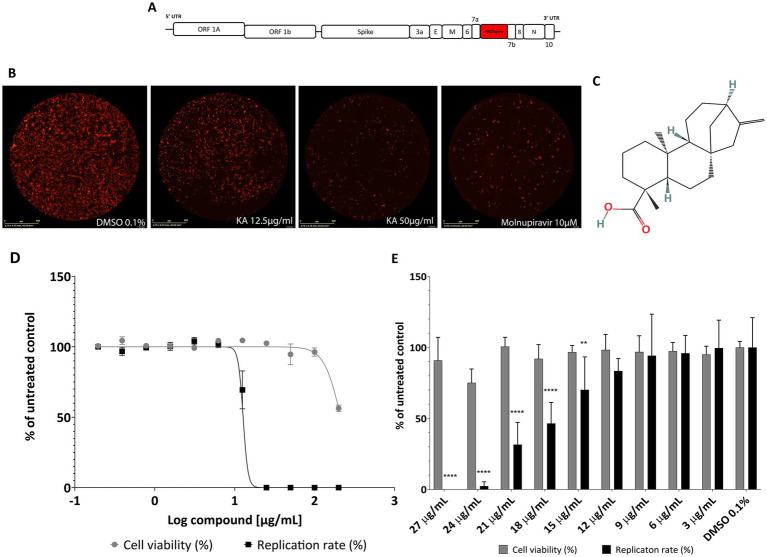
KA inhibits SARS-CoV-2-mCherry replication in A549-AT cells with a high selectivity index. **(A)** Schematic representation of SARS-CoV-2-mCherry genome (drawing is not to scale), and **(B)** a representative image of red fluorescence in A549-AT cells after SARS-CoV-2-mCherry infection, treatment with KA at 12.5 and 50 μg/mL, and Molnupiravir at 10 μM. **(C)** Chemical structure of kaurenoic acid (KA) (PubChem CID: 73062). **(D)** Dose–response curve of KA against SARS-CoV-2 replication and A549-AT viability. **(E)** Antiviral screening of KA in a smaller range of concentrations, ranging from 3 to 27 μg/mL. SARS-CoV-2 replication was measured at 24-h post-infection by calculating the total integrated intensity of the red fluorescence and cell viability by MTT assay; values obtained for KA-treated samples were normalized to those of DMSO 0.1% treated samples. The mean values and standard deviation (SD) of three independent experiments, each performed in four technical repeats. **p < 0.01 ****p < 0.0001 (one-way ANOVA).

To assess the effect of KA on A549-AT viability and SARS-CoV-2 replication, cells were treated with 2-fold serial dilutions of KA in the absence or presence of recombinant SARS-CoV-2-expressing the mCherry fluorescence reporter (SARS-CoV-2-mCherry) (Figure 1A). The fluorescence intensity of cells infected with SARS-CoV-2-mCherry is proportional to virus replication (Figure 1B). Interestingly, KA impaired viral replication in 12.5- and 50-μg/mL concentrations, as shown by the decrease in fluorescence. We employed Molnupiravir at 10 μM as a positive inhibition control, in which similar fluorescence levels between Molnupiravir and KA inhibition were observed (Figure 1B). Therefore, a dose–response curve was performed to characterize KA’s viral inhibition and cytotoxicity (Figure 1C). As a result, KA had a CC_50_ and EC_50_ of 212.9 μg/mL (703.8 μM) and 13.2 μg/mL (43.6 μM), respectively (Figure 1D), with a calculated selective index (SI) of 16.1.

### 3.2 KA does not inhibit SARS-CoV-2 entry

**Figure 2:**
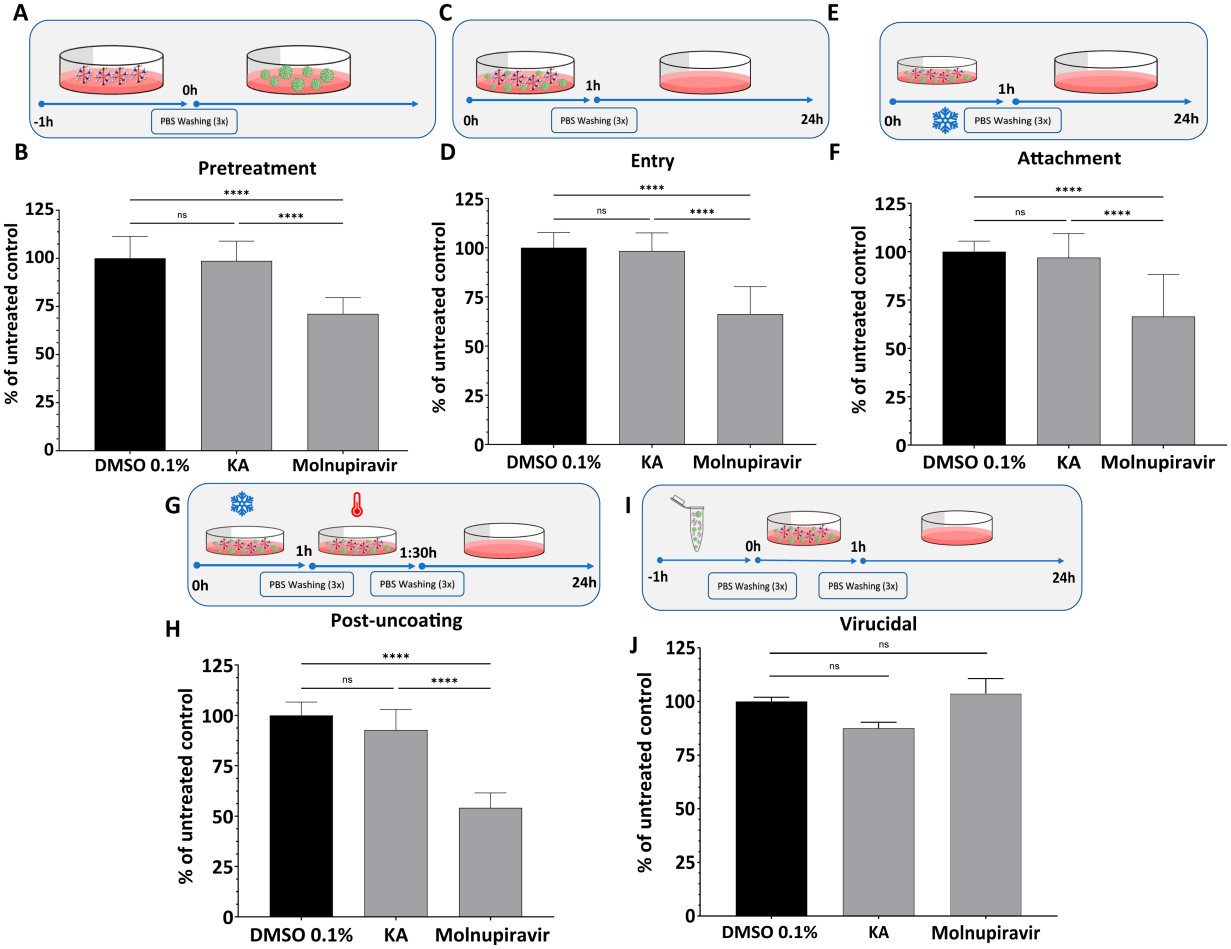
KA does not inhibit early steps of SARS-CoV-2-mCherry infection. The effect of kaurenoic acid (KA) on cell pretreatment **(A,B)**, SARS-CoV-2-mCherry entry **(C,D)**, attachment **(E,F)**, uncoating **(G,H)**, and virucidal **(I,J)** is shown. For each assay, the values of integrated intensity of fluorescence in KA-treated cells were normalized to those from control cells. Molnupiravir at 10 μM was used as a positive control. SARS-CoV-2 replication was measured at 24-h post-infection by calculating the total integrated intensity of the red fluorescence; values obtained for KA-treated samples were normalized to those of DMSO 0.1% treated samples. The mean values from three independent experiments, each performed in quadruplicate, and the standard deviation (SD) are shown. ns, non-significant, ****p < 0.0001 (one-way ANOVA). DMSO, dimethyl sulfoxide; PBS, phosphate-buffered saline; UTR, untranslated region. Incubation at 4° C is indicated by the snowflake, and 37° by the red thermometer.

Due to the inhibition profile of KA, we continued to characterize its effect by screening it on different steps of the SARS-CoV-2 replicative cycle. To this, we performed a set of assays employing the inhibitor at 25 μg/mL, a concentration resulting in near-complete inhibition of SARS-CoV-2 replication (Figures 1D,E). Pre-treatment of the cells with KA (Figure 2A) revealed that the compound was not able to protect them against SARS-CoV-2 infection (Figure 2B). Furthermore, to analyze the impact of KA on the early steps of SARS-CoV-2 infection, three different treatments were performed: (1) an entry assay; (2) an attachment assay; and (3) an internalization assay. In none of the treatments KA was able to impair SARS-CoV-2 infection (Figures 2C–H). Furthermore, a virucidal assay (Figure 2I) resulted in a similar outcome, with no inhibition of SARS-CoV-2-mCherry (Figure 2J). In contrast, Molnupiravir reduced at least 25% of viral replication in all entry assays, besides the virucidal (Figures 2A–J). Combined, these data suggest that KA might inhibit the post-entry steps of SARS-CoV-2 infection.

### 3.3 KA strongly inhibits the post-entry step of SARS-CoV-2 infection

**Figure 3:**
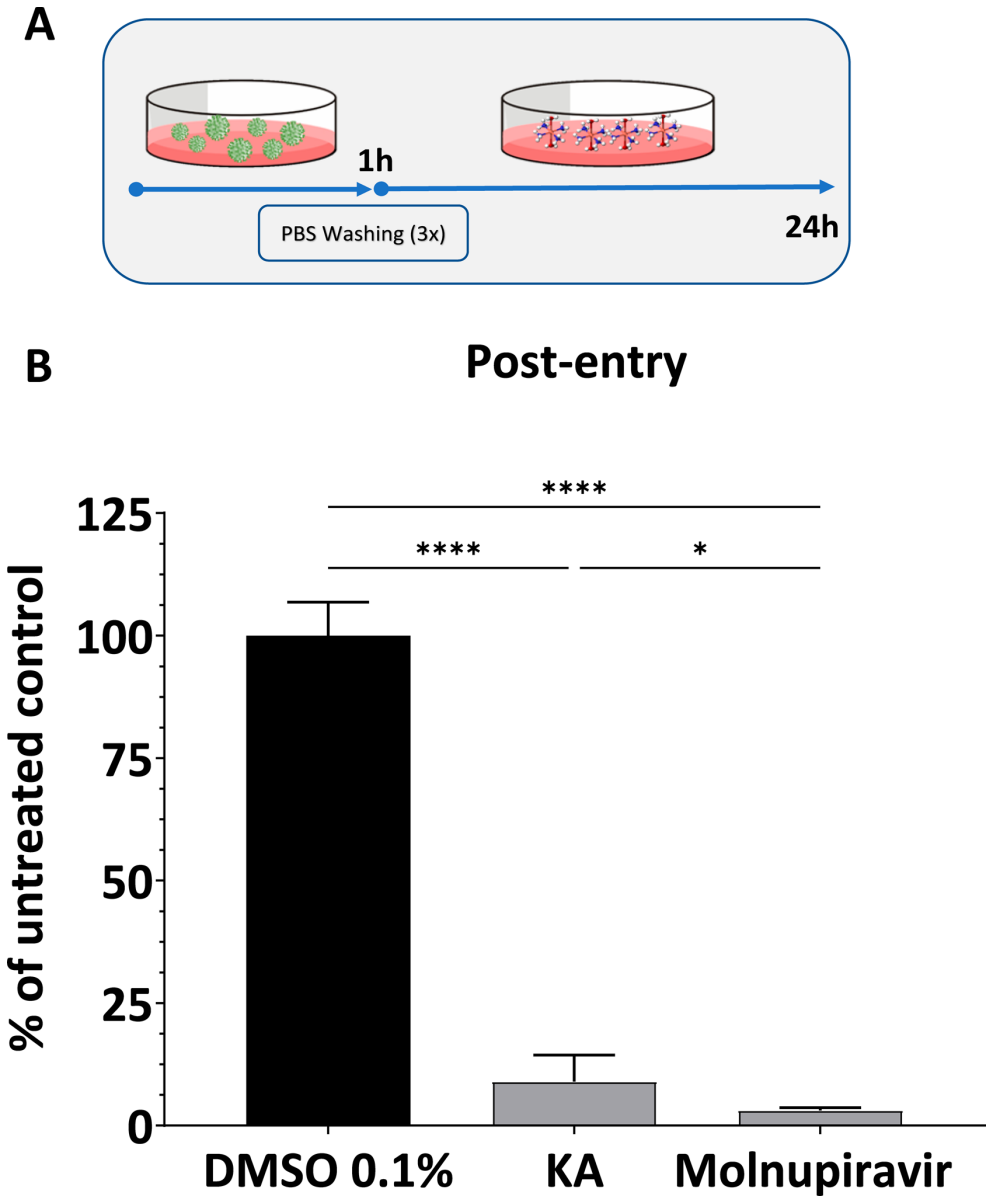
KA inhibits post-entry steps of SARS-CoV-2 infection. **(A)** Schematic representation of post-entry assay. **(B)** Effect of kaurenoic acid (KA) at 25 μg/mL in SARS-CoV-2-mCherry post-entry. SARS-CoV-2 replication was measured at 24 h post-infection by measuring the total integrated intensity of the red fluorescence; values obtained for KA-treated samples were normalized to those of DMSO 0.1% treated samples. Molnupiravir at 10 μM was used as a positive control. Mean values ± standard deviation (SD) of a minimum of three independent experiments, each performed in triplicate, are shown. ns, non-significant, *p < 0.05, ^****^p < 0.0001 (one-way ANOVA). DMSO, dimethyl sulfoxide; PBS, phosphate-buffered saline.

Since KA failed to inhibit the early steps of SARS-CoV-2 infection, its impact on post-entry steps was analyzed by infecting cells with SARS-CoV-2 and treating them with KA (Figure 3A). In this assay, treatment with KA inhibited viral replication by 91.1% (Figure 3B), confirming that the main activity of this molecule is due to targeting steps after viral entry into the cells. Similarly, Molnupiravir inhibited 95% of viral replication (Figure 3B).

**Figure 4:**
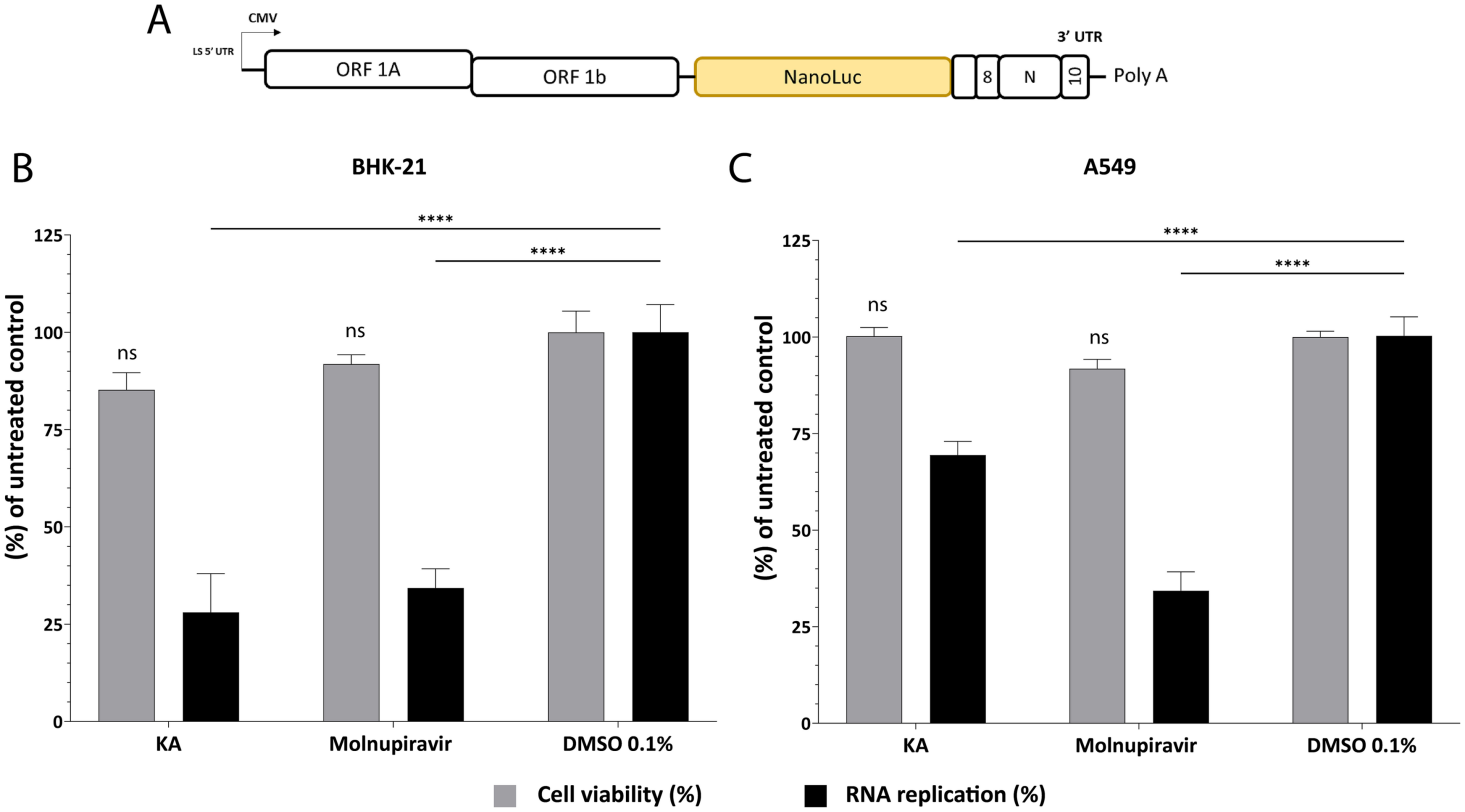
KA inhibits SARS-CoV-2 RNA synthesis in BHK-21 and A549-AT cells. Schematic representation of SARS-CoV-2-Repl-PL-NLuc subgenomic **(A)**. Kaurenoic acid (KA) at 25 μg/mL affects subgenomic RNA replication and cell viability in BHK-21 cells **(B)** and A549-AT cells **(C)**. SARS-CoV-2 subgenomic replication was measured at 72-h post-transfection by measuring the luminescence levels; values obtained for KA-treated samples were normalized to those of DMSO 0.1% treated samples. Molnupiravir at 10 μM was used as a positive control. Mean values ± standard deviation (SD) of three biological repeats are shown. ^****^p < 0.0001 (one-way ANOVA). CMV, cytomegalovirus promoter; DMSO, dimethyl sulfoxide; NanoLuc, nanoluciferase.

For a more detailed analysis of the effect of KA on SARS-CoV-2 infection, we performed an assay employing the SARS-CoV-2 subgenomic replicon carrying the nanoluciferase (NanoLuc) marker (SARS-CoV-2-Repl-PL-NLuc, Figure 4A). Through this assay, it is possible to investigate RNA replication/translation without forming viral particles. We first confirmed that KA was not cytotoxic in BHK-21 cells as these cells were used for the SGR assays and would be treated longer than the infectious virus assays. Reassuringly, KA did not show cytotoxicity at concentrations lower than 10 μg/mL (Supplementary Figure 3). When BHK-21 cells were transfected with the SARS-CoV-2-Repl-PL-NLuc cDNA plasmid and treated with KA, subgenomic replication was reduced by 62%, while Molnupiravir treatment reduced it by 63% (Figure 4B). To confirm if the effect observed in BHK-21 cells was not cell-dependent, the same protocol was carried out using A549-AT cells. Interestingly, KA was also not cytotoxic at 10 μg/mL, similar to BHK-21 cells (Supplementary Figure 4). The treatment in transfected A549-AT cells reduced replication of the subgenomic replicon by 31% while the positive control reduced by 64% (Figure 4C), demonstrating that it inhibits viral RNA replication and/or synthesis of subgenomic RNAs.

**Figure 5:**
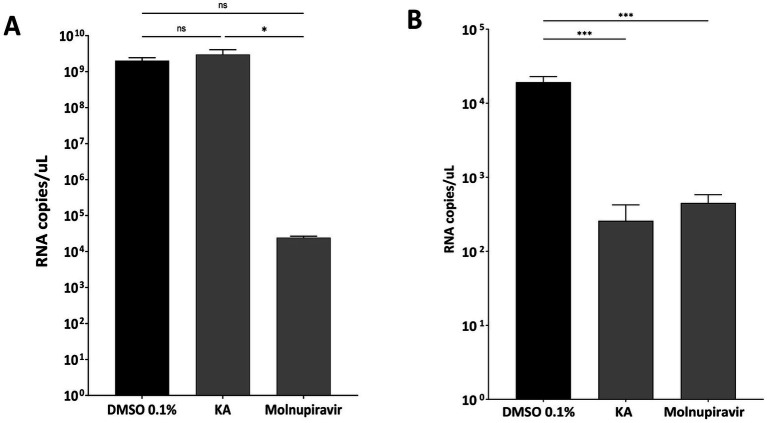
KA inhibits assembly and release of SARS-CoV-2 virions. Copy numbers of viral genomic RNAs in lysates **(A)** and supernatants **(B)** of SARS-CoV-2 wild-type (Wuhan-like virus) after kaurenoic acid (KA) (25 μg/mL) treatment are shown in Log_10_ RNA copies/mL. Molnupiravir at 10 μM was used as a positive control. Mean values ± standard deviation (SD) of three independent experiments. ^*^p < 0.05; ^***^p < 0.001 (Student’s *t*-test). DMSO, dimethyl sulfoxide.

We performed an additional analysis to understand if viral inhibition was related to RNA replication/translation or further downstream into post-entry steps of SARS-CoV-2 infection. Since virion formation and release are absent in subgenomic replicon-transfected cells, we infected A549-AT cells with SARS-CoV-2 Wuhan-like strain (hCov-119/England/02/2020) using the protocol shown in Figure 3A. We quantified the viral mRNA levels in the supernatant and in cell lysates using RT-qPCR. As a result, in this experiment, levels of viral RNA in KA-treated cell lysates were similar to those in mock-treated cells. Therefore, significant inhibition of RNA synthesis was not observed (Figure 5A). In contrast, KA treatment reduced the levels of viral genomic RNA in the supernatants by 91.8% (Figure 5B), suggesting that in A549-AT cell culture, the compound also impairs SARS-CoV-2 virion assembly and/or release. this looks to be in bold, please check and make it similar to the text both intracellular and supernatant (Figures 5A,B), demonstrating that KA has a different effect on the viral replicative cycle than the control.

### 3.4 KA inhibits Delta (B.1.617.2) and Omicron (BA.2) variants of SARS-CoV-2

**Figure 6:**
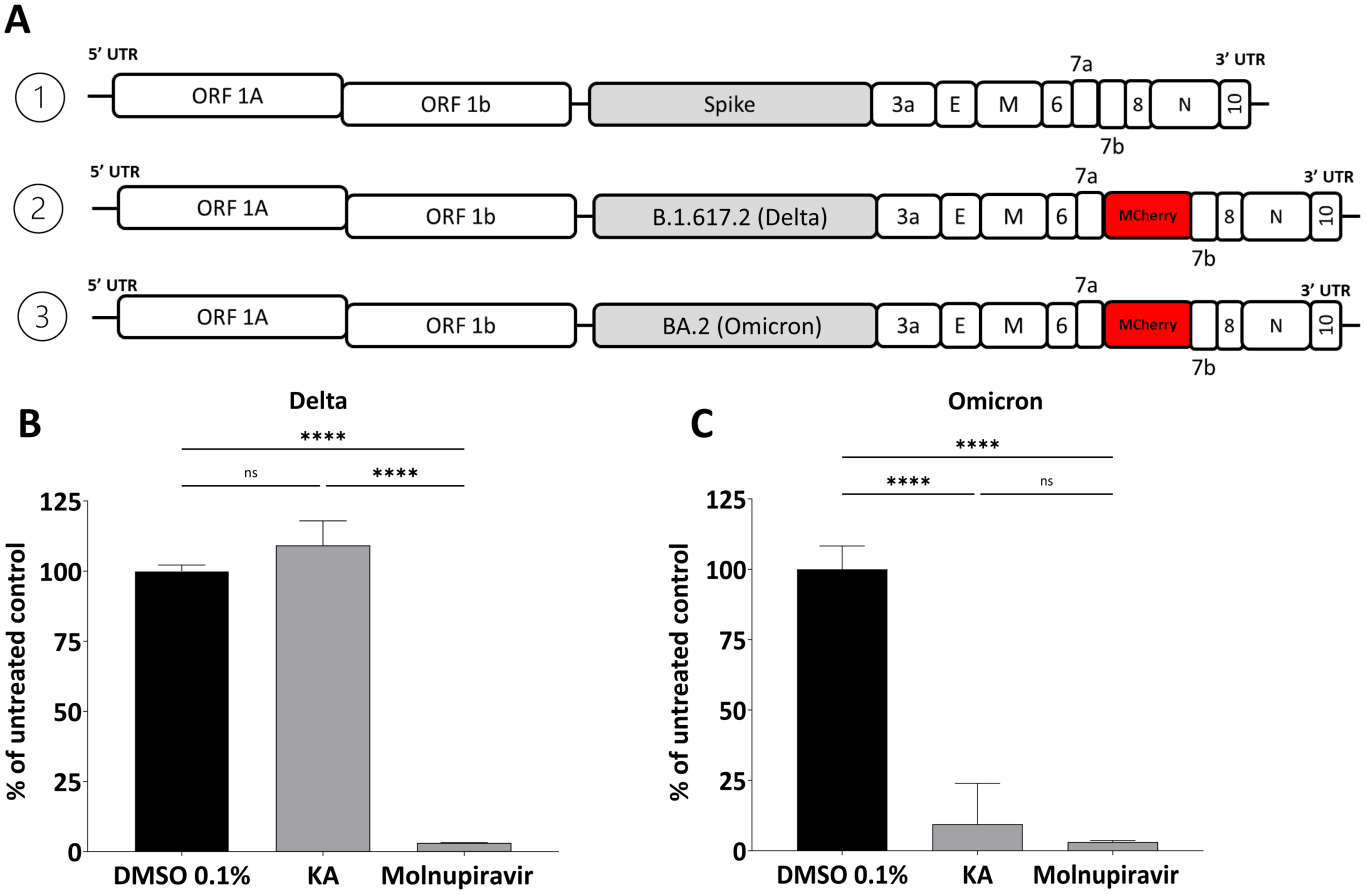
Effect of KA on chimeric SARS-CoV-2 variants. **(A)** Schematic representation of the genomes of SARS-CoV-2-Wuhan-Delta-Spike and SARS-CoV-2-Wuhan-Omicron-Spike chimeras. The effect of kaurenoic acid (KA) at 25 μg/mL on SARS-CoV-2-Wuhan-Delta-Spike **(B)** and SARS-CoV-2-Wuhan-Omicron-Spike **(C)** is shown. SARS-CoV-2 replication was measured at 24 h post-infection by measuring the total integrated intensity of the red fluorescence; values obtained for KA-treated samples were normalized to those of DMSO 0.1% treated samples. Molnupiravir at 10 μM was used as a positive control. Mean values ± standard deviation (SD) of a minimum of three independent experiments, each performed in triplicate, are shown. ^****^p < 0.0001 (one-way ANOVA). DMSO, dimethyl sulfoxide.

Due to the emergence of new variants of SARS-CoV-2 with an accumulation of mutations in different parts of the genome, we performed assays with the main VOCs to audit the KA effect on other variants. First, as S-protein is crucial for SARS-CoV-2 virion formation and release, we assessed the effect of KA against the Delta and Omicron S-protein chimeras constructed using an infectious clone of the Wuhan strain. For this, the spike gene of SARS-CoV-2-mCherry was replaced with its counterpart from the Delta (B.1.617.2) or Omicron (BA.2) variants (Figure 6A). Unexpectedly, by the quantification of mCherry fluorescence, it was observed that KA had a minimal impact against the chimeric virus harboring the Delta-Spike (Figure 6B) while at the same time, replication of the virus harboring the Omicron Spike was inhibited by 90.5% (Figure 6C). Molnupiravir inhibited above 95% of both chimeric viruses (Figures 6B,C).

**Figure 7:**
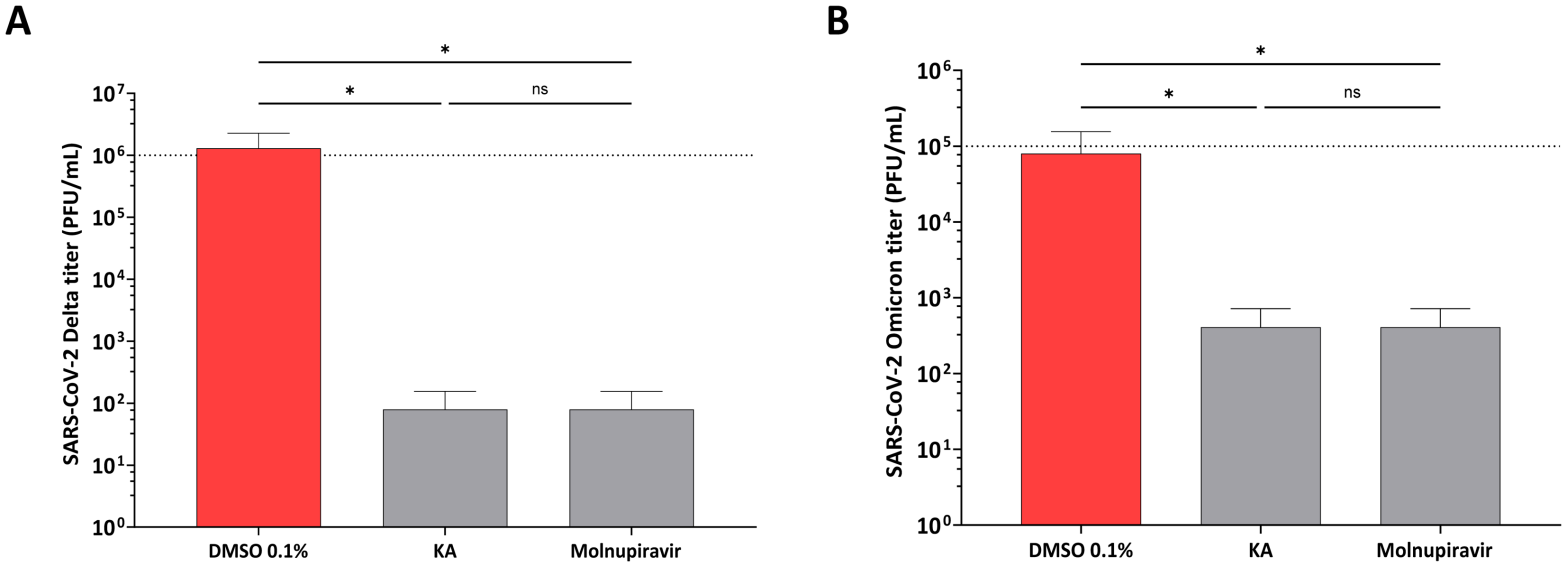
KA reduces titers of SARS-CoV-2 Delta and Omicron variants. A549-AT cells were infected with SARS-CoV-2 Delta **(A)** or Omicron **(B)** variants in the presence of kaurenoic acid (KA) at 25 μg/mL, and 24 h virus titers are shown. Molnupiravir at 10 μM was used as a positive control. After 24h, supernatants were collected and virus titers were determined by the TCID_50_ method. Mean values ± standard deviation (SD) of three independent experiments, each measured in triplicate, are shown. ^*^p < 0.05 (one-way ANOVA). DMSO, dimethyl sulfoxide; PFU/mL, plaque-forming units per milliliter.

Furthermore, the effect of KA was confirmed by employing the wild-type SARS-CoV-2 isolates representing Delta (B.1.617.2) and Omicron (BA.2) variants. The experiment was performed as described above, except that the impact of the compound on virus replication was analyzed by comparing the titers of released infectious viruses. In contrast to the previous experiment, it was observed that KA treatment resulted in a decrease in the titers of both variants, being the effect on the Delta variant (4 log_10,_ decrease of titer Figure 7A) higher than on the Omicron variant (3log_10_ decrease of titer, Figure 7B), but similar titers to Molnupiravir treated samples.

### 3.5 KA potentially binds to SARS-CoV-2 proteases

**Figure 8:**
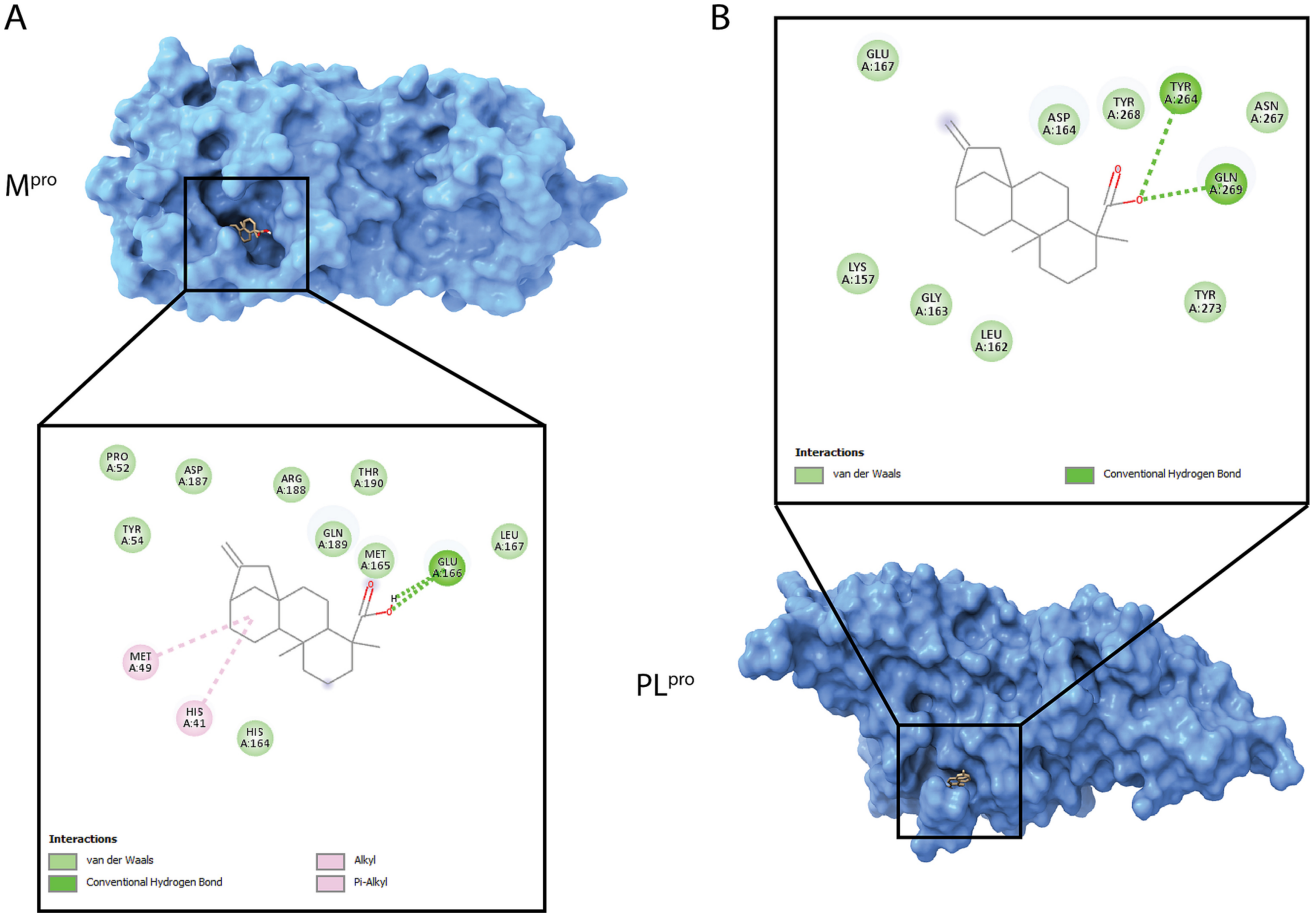
3D and 2D visualization of the SARS-CoV-2 proteases with KA docking analyses. **(A)** Best scored main protease (M^pro^, blue) docking pose with the molecule. **(B)** Best scored of papain-like protease (PL^pro^, blue) docking pose with the molecule. 3D protein is shown in surface and ligands as sticks. The two-dimensional (2D) diagram shows the main atom interactions between the ligand and the protein.

Considering our results gathered above, we performed extensive research into kaurene molecules, and interestingly, kaurene diterpenoid analogs to KA have been proposed to interact with various SARS-CoV-2 proteins, including the main protease (3CL^pro^ or M^pro^) and the papain-like protease (PL^pro^) (Nurlela et al., 2022). Given the significant post-entry effects and the reduction in viral release, we hypothesized that one of these proteins could be a target of this molecule. Therefore, we conducted molecular docking studies between KA and M^pro^ and PL^pro^, revealing that KA can bind to both proteases (Figure 8).

In M^pro^, KA bound to catalytic dyad through an alkyl bond to His41, hydrogen bond with Glu166, and van der Waals interactions with Pro52, Tyr54, His164, Met165, Leu167, Asp187, Arg188, Gln189, and Thr190 resulting into a CHEM-PLP score of 78.62, binding energy of −6.273 kcal/mol and ligand efficiency of −0.2851 kcal/mol (Figure 8A). In contrast, KA bound to PL^pro^ in the substrate pocket site with hydrogen bonds to TYR264 and GLN269 as well as van der Waals interactions with Lys157, Leu162, Gly163, Asp164, Glu167, Asn267, Tyr268, and Tyr273 (Figure 8B), which was calculated into a CHEM-PLP score of 91.91, binding energy of −6.406 kcal/mol, and ligand efficiency of −0.2912 kcal/mol.

## 4 Discussion

The global battle against SARS-CoV-2 demands the continuous search for novel compounds with the potential to mitigate its impact. In this study, we evaluated the efficacy of KA, a molecule with the potential to be employed in pre-clinical trials, against SARS-CoV-2 infection. Even though the effect of this compound against parasites such as *Trypanosoma* and *Leishmania* species (Kian et al., 2018) is well described, only one study demonstrated through molecular docking calculations that KA could be an inhibitor of SARS-CoV-2 by interacting with the Spike protein (Santos W. et al., 2021). Our study describes and characterizes the antiviral activity of KA in cell culture against SARS-CoV-2 proteases. As a result of the low cytotoxicity, we found that the SI of KA against SARS-CoV-2 was 16.1, considerably higher than the SI against *Leishmania infantum* and *Leishmania amazonensis* (5.3 and 5.19, respectively) (Kian et al., 2018). Furthermore, KA was previously described with low cytotoxicity in HEPG2 cells (CC_50_ of 268.7 μg/mL), agreeing with our data in which the treatment resulted in low cytotoxicity to A549-AT cells (CC_50_ of 212 μg/mL) and demonstrates the tolerance of this compound among different cell lines. In addition, other molecules isolated from natural sources were described with inhibitory activity against SARS-CoV-2, but with an SI between 2 and 10, which further emphasizes KA as a potent compound (Herzog et al., 2024).

The analysis of the KA mechanism of action using different assays revealed that the compound had no virucidal effect and was unable to inhibit SARS-CoV-2 entry. In contrast, KA had a high impact in the SARS-CoV-2 post-entry stages. Interestingly, other extracts and compounds isolated from NPs have been described as affecting post-entry steps of SARS-CoV-2 replication by interacting with host cell responses or directly with viral proteins (Herzog et al., 2024). In this study, different parameters were used to evaluate the mechanism of action of KA. The first parameter involved using a subgenomic replicon to monitor the synthesis of subgenomic RNA, while the second parameter involved infectious virus experiments to measure the synthesis of full-length genomic RNA. In all assays employed, KA decreased viral replication in the maturation and release steps, even in different VOCs. Previous data showed that isolated molecules were able to inhibit different SARS-CoV-2 VOCs, and together with our results, it further emphasizes the unique architecture that plays an essential role in fighting pathogens (Santos et al., 2020; Chuong et al., 2021; Nurlela et al., 2022; Uppal et al., 2022; Grosche et al., 2023).

KA was able to block the release of infectious virions of the SARS-CoV-2 Delta variant, while it did not affect mCherry expression (which occurs from subgenomic RNA) in experiments performed using SARS-CoV-2-Wuhan-Delta-Spike. As such, we may hypothesize that in the native context, the S-protein of the Delta variant causes high susceptibility to KA. In contrast, in the context of the Wuhan strain containing the Delta S-protein, it results in resistance. Additionally, this can be further emphasized by the capacity of the S-protein of Delta to be more fusogenic and form syncytia in cell culture (Saito et al., 2022), which could interfere with the fluorescence measurements in the assay by allowing mCherry signal from infected cells to spread across the cell layer, independently of infectious virion release. Since total fluorescence in each well was measured, rather than contagious virion release alone, the assay might underestimate KA’s effect on viral release specifically, posing a limitation of the technique employed here. This limitation was reported before with the GFP reporter gene, where complemented GFP signals did not increase significantly over time among cells with syncytia formation, compared to nanoluciferase signals (Wang et al., 2022), showing that infection is occurring, but fluorescence signals can have a delay. Furthermore, the employment of viruses carrying different reporter genes could also help in assessing the viral replication and not be impacted by the syncytia formation (Chiem et al., 2022).

Undoubtedly, KA can impair SARS-CoV-2 replication by significantly disrupting the assembly and/or release of SARS-CoV-2 virions. Interestingly, KA interaction with SARS-CoV-2 S protein was exploited in molecular docking calculations, demonstrating some stability with alkyl, pi-alkyl, H-bond, and Van der Waals interactions with negative binding energy (Santos W. et al., 2021). In our study, the data support the hypothesis that the KA effect is related to other proteins besides S, since no entry inhibition was observed in the antiviral assays performed. Interestingly, an *in silico* assay with kaurene diterpenoids analogs against the M^pro^, PL^pro^, nucleocapsid (N), and membrane (M) proteins from SARS-CoV-2, revealed that kaurene derivatives evaluated were interacting with the proteases with low values of Gibbs free energy, which might result in reduced SARS-CoV-2 assembly (Nurlela et al., 2022). Here, our data shows a higher score and lower binding energy in molecular docking with PL^pro^, an important polyprotein processing enzyme essential for the maturation and formation of the replicase-transcriptase complex (RTC) (Shin et al., 2020). The PL^pro^ cleaves proteins after the substrate LXGG sequence inside the substrate binding pocket, which is the region where KA was shown to interact, and therefore, can be a competitive inhibitor of the substrate. Notably, natural products were previously identified as reversible inhibitors of PL^pro^, among those, tanshinones from *Salvia miltiorrhiza*, chalcones and coumarins from *Angelica keiskei*, and polyphenols from *Broussonetia papyrifera* (Wasilewicz et al., 2023). In another study, the inhibition of PL^pro^ resulted in low levels of SARS-CoV-2 subgenomic replication and decreased the release of infectious virus into the supernatant (De Oliveira et al., 2020). This agrees with our data, in which we demonstrate that KA treatment decreased the SARS-CoV-2 titer in supernatant without impacting viral genomic RNA levels in cytoplasm is consistent with the hypothesis that the effect of the compound on the virion assembly and release may be due to its interaction with PL^pro^, and since this protein is highly conserved among variants would explain the effect against them. Regardless of whether this is the case or not, our data obtained using natural Delta and Omicron variants clearly shows that the target of KA is not affected by the mutations accumulated in these SARS-CoV-2 variants.

Another aspect of the potential therapeutic potential of KA is its safety profile. The pharmacokinetics and oral bioavailability of KA in Wistar rats were previously evaluated (Matos et al., 2018), showing that the compound possessed satisfactory stability and was widely distributed in fluids and organs intravenously administered at 50mg/kg. The ability of compounds to traverse cell membranes is consistent with our data, which suggests that it strongly affects the post-entry stages of SARS-CoV-2 infection. KA was described as an anti-inflammatory agent in animal models, recovering the nitric oxide pathway, inducing IL-1β, and upregulating NLRP12 in *L. amazonensis*-infected macrophages treated with KA. These, in association with our findings of KA effect on SARS-CoV-2 replication, are a combination that is particularly significant considering the pathogenesis of COVID-19, with the disease resulting in increased IL-6 and other inflammatory cytokines in the patients’ blood (Miranda et al., 2015; Cao and Li, 2020). Therefore, administering this compound *in vivo* may contribute to enhanced recovery by viral clearance and anti-inflammatory mechanisms.

## 5 Conclusion

Kaurenoic acid (KA) is a novel inhibitor of SARS-CoV-2 replication with an SI of 16.1. KA specifically impairs post-entry steps of SARS-CoV-2 replicative cycle by decreasing the RNA replication and viral release by possibly interacting with its proteases PL^pro^ and M^pro^ substrate pocket, disrupting virion assembly and release. Importantly, this effect remains consistent across different SARS-CoV-2 isolates, indicating the potential for future emergent variants. Altogether, this study demonstrates KA as a promising drug against SARS-CoV-2.

## Data Availability

The raw data supporting the conclusions of this article will be made available by the authors, without undue reservation.
