# Supplementary material for: Kaurenoic acid is a potent inhibitor of SARS-CoV-2 RNA synthesis, virion assembly, and release in vitro
Source: Front Microbiol. 2025 May 9;16:1540934. doi: 10.3389/fmicb.2025.1540934 (PMC12098342; doi:10.3389/fmicb.2025.1540934)
Supplement: Supplementary file 1 [file Data_Sheet_1.docx]

Supplementary Material

## Supplementary Figures


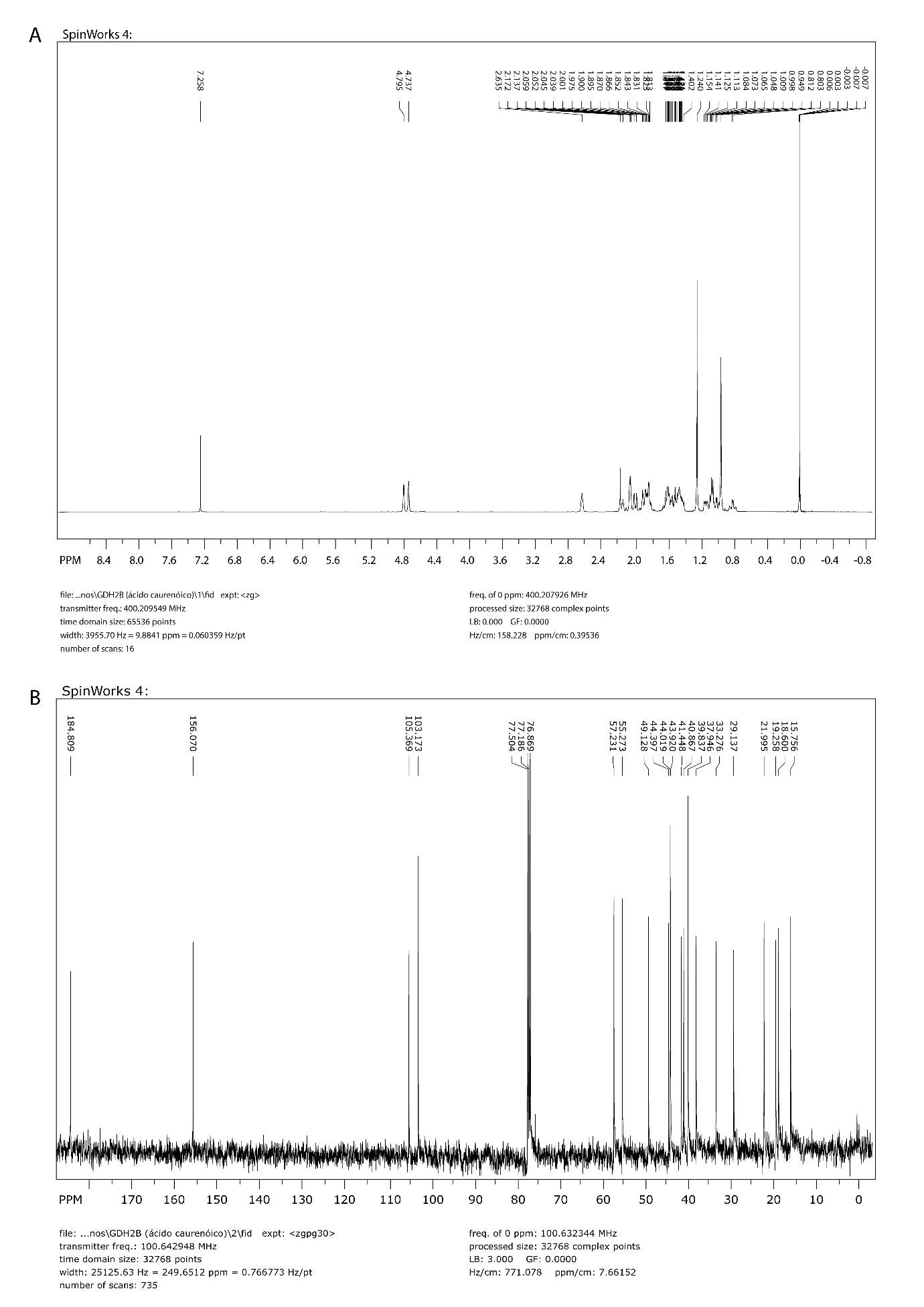


**Supplementary figure 1:** ^1^H-NMR (A) and ^13^C-NMR (B).

**
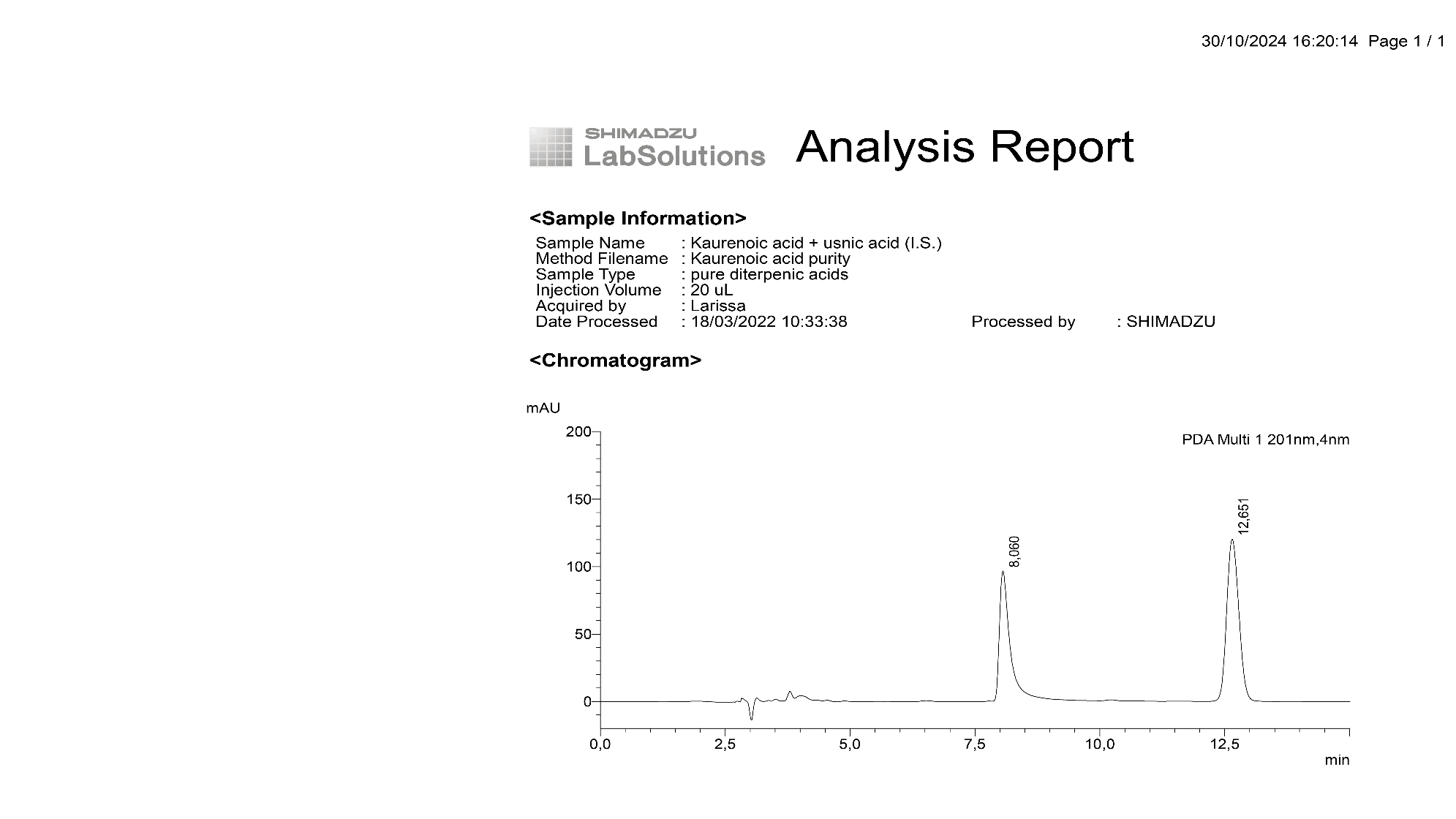
**

**Supplementary figure 2:** KA and usnic acid (standard) on HPLC-DAD.

**Supplementary Figure 3. Cell viability of KA in BHK-21 cells**. BHK-21 cells were treated with KA at three different concentrations. Cell viability was measured by the MTT assay 72 h after treatment.

**Supplementary Figure 4. Cell viability of KA in A549-AT cells**. A549-AT cells were treated with KA at three different concentrations. Cell viability was measured by the MTT assay 72 h after treatment.
